# Determining optimal clinical target volume margins based on microscopic tumor extension in patients with non-small-cell lung cancer undergoing chemotherapy or chemotherapy combined with immunotherapy

**DOI:** 10.3389/fonc.2025.1503615

**Published:** 2026-01-12

**Authors:** Yujiao Zhang, Xiao Song, Jiaran Li, Fen Zhao, Li Li, Ning Liu, Miaoqing Zhao, Shuanghu Yuan

**Affiliations:** 1 Department of Oncology, Affiliated Qingdao Central Hospital of Qingdao University, Qingdao Central Hospital, University of Health and Rehabilitation Sciences, Qingdao, Shandong, China; 2 Department of Radiation Oncology, Shandong Cancer Hospital and Institute, Shandong First Medical University and Shandong Academy of Medical Sciences, Shandong Cancer Hospital Affiliated to Shandong First Medical University, Jinan, Shandong, China; 3 Department of Pathology, Rongcheng People′s Hospital, Weihai, Shandong, China; 4 Department of Radiation Oncology, The First Affiliated Hospital of USTC, Division of Life Sciences and Medicine, University of Science and Technology of China, Hefei, Anhui, China; 5 Department of Pathology, Shandong Cancer Hospital and Institute, Shandong First Medical University and Shandong Academy of Medical Sciences, Jinan, Shandong, China; 6 Department of Radiation Oncology, Shandong Cancer Hospital and Institute, Shandong First Medical University and Shandong Academy of Medical Sciences, Jinan, Shandong, China

**Keywords:** chemotherapy, chemotherapy combined with immunotherapy, clinical target volume, microscopic tumor extension, non-small-cell lung cancer

## Abstract

**Background:**

There is currently no unified understanding of the clinical target volume (CTV) margins for radiotherapy in non-small cell lung cancer (NSCLC), after chemotherapy or chemotherapy combined with immunotherapy. This study aimed to explore margins around the gross tumor volume (GTV) to infer the CTV in NSCLC, after chemotherapy or chemotherapy combined with immunotherapy, using microscopic tumor extension (ME).

**Methods:**

We retrospectively analyzed 185 patients with stage II and III NSCLC who underwent surgery without neoadjuvant therapy or with neoadjuvant chemotherapy or chemotherapy combined with immunotherapy. We assessed the correlation between the radiological (last preoperative computed tomography image) and pathological (postoperative gross specimens) tumor size, measured the distance of ME under a digital microscope, and determined the correlation between clinicopathological characteristics and ME.

**Results:**

For the same tumor sample, a significant correlation was observed between the radiological and macroscopic sizes (r=0.836). With a 5% error risk, we applied a margin of 6.80 mm and 6.00 mm to the adenocarcinoma (ADC) and squamous cell carcinoma (SCC) of direct surgery, respectively; a margin of 5.20 mm and 4.20 mm, to the ADC and SCC receiving chemotherapy, respectively; and a margin of 4.60 mm and 1.20 mm to the ADC and SCC receiving chemotherapy combined with immunotherapy, respectively. The ME value of a tumor correlated with the pathological type, tumor stage, and preoperative treatment methods.

**Conclusions:**

Different CTV margins should be adopted for patients with NSCLC who are receiving different treatments.

## 1 Introduction

Lung cancer has the second-highest incidence rate and the highest mortality among all cancers worldwide (1), with non-small cell lung cancer (NSCLC) accounting for approximately 85% of all lung cancer cases (2). Radiotherapy has become one of the primary treatment modalities for NSCLC. The main mechanism of radiotherapy is that ionizing radiation directly or indirectly (through reactive oxygen species) damages Deoxyribonucleic acid, triggering a series of events that may lead to cell death (3). However, due to the non selective killing effect of radiotherapy on cells (3), if it extends too much outward on the base of gross tumor volume (GTV), it will increase the irradiation volume of healthy tissue, thereby increasing the risk of adverse reactions. Therefore, in radiotherapy, the precision of the target volume delineation greatly affects the safety and effectiveness of this treatment, especially the limits of the clinical target volume (CTV) (4). Giraud et al. (4) quantitatively evaluated the microscopic tumor extension (ME) of NSCLC by conducting a pathological analysis of specimens, after surgery from patients with NSCLC who underwent direct surgery (DS). Their findings suggest that the CTV of lung adenocarcinoma (ADC) and squamous cell carcinoma (SCC) should be extended outward by 8 mm and 6 mm, respectively, based on the GTV (4).

Recently, with the advancement of cancer research, preoperative neoadjuvant therapy has become the standard treatment method for operable NSCLC. Surgery after neoadjuvant chemotherapy (NAC) provides better survival benefits than does direct surgical treatment (5–9). Additionally, with the rise in immunotherapy, several recent studies have demonstrated that patients receiving neoadjuvant chemotherapy combined with immunotherapy (NACI) have a more significant survival benefit than that of those receiving NAC (10–12). However, some patients undergoing neoadjuvant therapy do not undergo surgical treatment for various reasons and choose to undergo radiotherapy. For inoperable NSCLC, sequential chemotherapy-radiotherapy has better therapeutic effects than radiotherapy alone (13, 14). While the optimal timing and strategy for immunotherapy intervention require further investigation and validation, several studies have preliminarily confirmed the feasibility of the early immunotherapy application (15, 16). Nevertheless, no studies have so far defined specific margins of GTV expansion for patients with NSCLC who receive chemotherapy or chemotherapy combined with immunotherapy followed by radiotherapy; thus, there is no clear optimal treatment plan. If these patients use the same CTV margin, it may increase their treatment toxicity and affect their quality of life, which is also contrary to the current personalized and precise treatment strategy.

Therefore, this study aimed to use postoperative anatomopathological slices to evaluate the expansion of NSCLC beyond the macroscopic visible tumor after NAC and NACI, so as to determine the optimal CTV margin for patients receiving chemotherapy or chemotherapy combined with immunotherapy.

## 2 Materials and methods

### 2.1 Patients

We conducted a retrospective analysis of patients with stage II and III NSCLC, who did not receive antitumor treatment before surgery and who received NAC or NACI before surgery at our institution from January 2022 to December 2023. We excluded patients who had previously received antitumor treatments and had pathological types other than ADC and SCC. We ultimately included 60 patients who did not receive anti-tumor therapy before surgery, 50 patients who received NAC before surgery, and 75 patients who received NACI before surgery. All patients underwent surgical resection either by pneumonectomy, lobectomy, segmental resection, or wedge resection based on tumor size and respiratory function. Pathological sections lacking sufficient normal tissues around the tumor body were excluded from the aforementioned patients, and 506 pathological sections (1 to 5 per patient) were finally included in the study. The patient characteristics of each group are provided in **Table 1**. All study procedures were performed in compliance with the Declaration of Helsinki and approved by the Ethical Review Board.

**Table 1:** Patient characteristics.

Characteristics	DS		NAC		NACI		Total
	ADC	SCC	ADC	SCC	ADC	SCC	
	n (%)	n (%)	n (%)	n (%)	n (%)	n (%)	n (%)
Patients	37 (20.00)	23 (12.43)	27 (14.59)	23 (12.43)	21 (11.35)	54 (29.19)	185 (100)
Slides	100 (19.76)	61 (12.06)	86 (17.00)	71 (14.03)	61 (12.06)	127 (25.10)	506 (100)
Age (mean)	61.49	65.87	60.22	63.17	58.67	63.07	62.20
Sex							
Male	24 (64.86)	18 (78.26)	17 (62.96)	21 (91.30)	15 (71.43)	50 (92.59)	145 (78.38)
Female	13 (35.14)	5 (21.74)	10 (37.04)	2 (8.70)	6 (28.57)	4 (7.41)	40 (21.62)
Smoking							
Yes	19 (51.35)	18 (78.26)	11 (40.74)	15 (65.22)	10 (47.62)	42 (77.78)	115 (62.16)
No	18 (48.65)	5 (21.74)	16 (59.26)	8 (34.78)	11 (52.38)	12 (22.22)	70 (37.84)
Clinical stage							
I	13 (35.14)	10 (43.48)	0 (0.00)	0 (0.00)	0 (0.00)	0 (0.00)	23 (12.43)
II	12 (32.43)	9 (39.13)	11 (40.74)	10 (43.48)	6 (28.57)	21 (38.89)	69 (37.30)
III	12 (32.43)	4 (17.39)	16 (59.26)	13 (56.52)	15 (71.43)	33 (61.11)	93 (50.27)
Pathological stage							
PCR	-	-	0 (0.00)	3 (13.04)	8 (38.10)	27 (50.00)	38 (20.54)
I	0 (0.00)	0 (0.00)	7 (25.93)	9 (39.13)	3 (14.29)	12 (22.22)	31 (16.76)
II	16 (43.24)	16 (69.57)	7 (25.93)	9 (39.13)	4 (19.05)	6 (11.11)	58 (31.35)
III	21 (56.76)	7 (30.43)	13 (48.15)	2 (8.70)	6 (28.57)	9 (16.67)	58 (31.35)

DS, direct surgery; NAC, neoadjuvant chemotherapy; NACI, neoadjuvant chemotherapy combined with immunotherapy; ADC, adenocarcinoma; SCC, squamous cell carcinoma; NS, no significance; PCR, pathologic complete response.

### 2.2 Pathology

**Figure 1:**
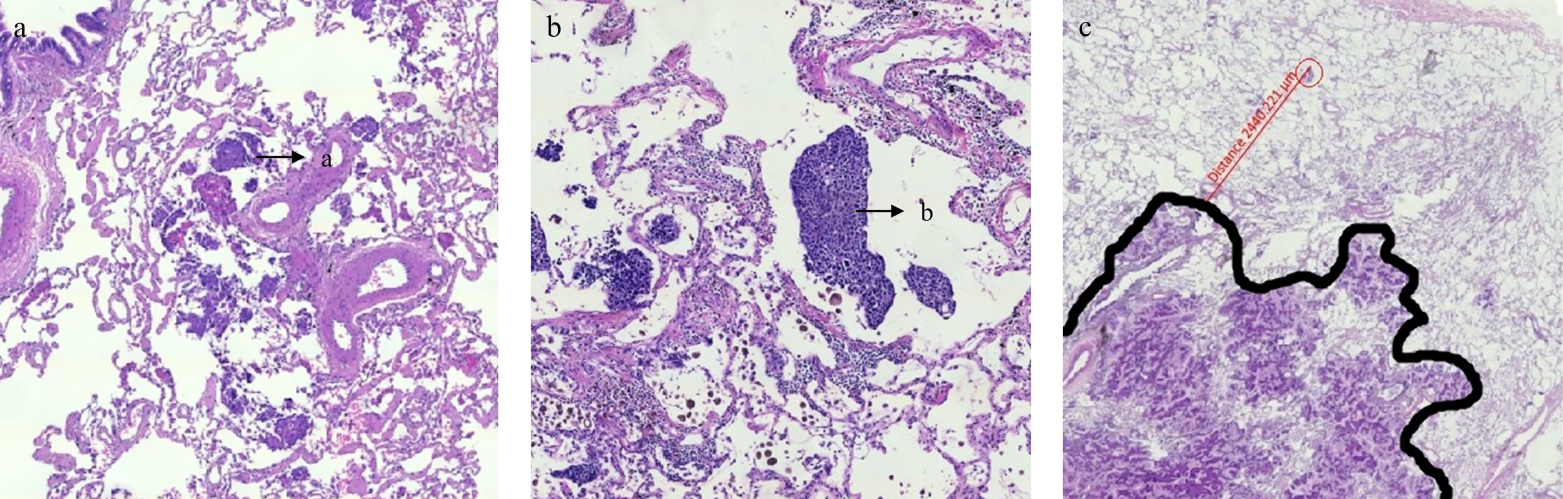
Metastatic tumor cells and measurement methods for microscopic tumor extension. **(a)** Microscopic tumor extension of adenocarcinoma (a: metastatic tumor cells). **(b)** Microscopic tumor extension of squamous cell carcinoma (b: metastatic tumor cells). **(c)** Measure the farthest microscopic extension from the boundary between the tumor and healthy tissue to metastatic tumor cells using a digital microscope.

Collect macroscopic tumor size and pathological tumor-node-metastasis (pTNM) staging data from the initial pathological report. Collect postoperative tumor specimens fixed in formalin, embedded in paraffin, and stained with hematoxylin eosin. Using ZEISS AxioScan.Z1 slide scanner (Carl Zeiss, Jena, Germany), the pathological sections were scanned, and Zeiss Zen 3.8 microscopy software (Carl Zeiss) was used to observe the pathological sections after scanning. Two groups of tumors were defined according to the 5th World Health Organization classification of thoracic tumors: ADC and SCC. To ensure study uniformity, other histological diagnoses were excluded owing to the small sample size. Subsequently, Zeiss Zen 3.8 microscopy software was employed to observe and determine microscopically extended tumor cells (as shown in **Figure 1A**, **Figure 1B** for ADC and SCC, respectively). The tumor cell with the farthest microscopic extension from the boundary, between the tumor and normal tissue, was selected to measure its ME distance (**Figure 1C**). For each slice, the longest ME was used for the final analysis.

### 2.3 Clinico-radiologic study

The clinical staging was determined according to the latest classifications. A radiologist measured the maximum diameter of each tumor on preoperative computed tomography.

### 2.4 Statistical analysis

Statistical analysis was performed using the Statistical Package for the Social Sciences version 25.0 (IBM Corp., Armonk, N.Y., USA). The comparison of qualitative parameters was conducted using χ^2^ test. An independent sample t-test or Mann–Whitney U-test was made to compare the differences between the two quantitative data samples. The relationship between the radiological and pathological size was evaluated using Spearman’s rank correlation. The ME of all samples were analyzed to determine the margin required for a sample recovery rate of 95%. Univariate and multivariate linear regression models were used to identify independent factors related to ME distance. Statistical significance was set at p < 0.05 significant.

## 3 Results

### 3.1 Patient and tumor characteristics

The basic characteristics of patients and tumors are provided in **Table 1**. In this study, we analyzed 100 slides made from 37 ADC and 61 slides made from 23 SCC in the DS group; 86 slides made from 27 ADC and 71 slides made from 23 SCC in the preoperative NAC group; and 61 slides made from 21 ADC and 127 slides made from 54 SCC in the preoperative NACI group. In general, the mean age of patients with SCC in each group was greater than that of patients with ADC. The proportion of male patients was significantly greater than female patients (78.38% vs. 21.62%), with male patients accounting for a larger proportion of patients in each group. Smokers constituted the majority (62.16% vs.37.84%), with a higher proportion of smokers with SCC than those with ADC. After neoadjuvant therapy, the pathologic complete response (PCR) rate of patients with SCC was significantly higher than that of patients with ADC (38.96% vs.16.67%; p=0.008).

The tumor sizes in each group of patients are shown in **Table 2**. Regarding radiography and pathology, the mean tumor size in the DS group was higher in patients with SCC than in those with ADC; however, the difference was statistically non-significant (p values = 0.90 and 0.16, respectively). The SCC of the NAC and NACI group were smaller than that of the ADC in both radiography and pathology; however, this difference lacked statistical significance (the p-values of the NAC group were 0.07 and 0.18, respectively; the p-values of the NACI group were 0.55 and 0.06, respectively).

**Table 2:** Tumor size and microscopic extension according to histology.

	n	Number of slides	Mean	Median	SD	Range
DS						
ADC radiologic size (cm)	37		3.49	3.40	1.50	1.20-7.00
SCC radiologic size (cm)	23		3.87	4.00	1.57	1.20-7.80
ADC pathological size (cm)	37		3.49	3.40	1.43	1.40-7.20
SCC pathological size (cm)	23		4.28	4.50	1.98	1.00-9.00
ADC ME (mm)	37	100	1.98	1.38	2.15	0-8.67
SCC ME (mm)	23	61	1.26	0.00	2.81	0-15.30
NAC						
ADC radiologic size (cm)	27		3.21	3.10	1.48	0.60-7.00
SCC radiologic size (cm)	23		2.64	2.30	1.83	0-8.90
ADC pathological size (cm)	27		3.04	2.50	1.36	1.20-6.50
SCC pathological size (cm)	23		2.68	2.50	1.91	0-9.00
ADC ME (mm)	27	86	1.58	0.79	1.86	0-6.66
SCC ME (mm)	23	71	0.84	0.00	2.05	0-13.70
NACI						
ADC radiologic size (cm)	21		3.32	2.80	1.56	1.10-6.50
SCC radiologic size (cm)	54		2.68	2.60	1.55	0-7.00
ADC pathological size (cm)	21		3.62	3.20	1.77	1.10-7.00
SCC pathological size (cm)	54		2.42	2.50	1.40	0-6.00
ADC ME (mm)	21	61	0.87	0.00	1.64	0-6.33
SCC ME (mm)	54	127	0.14	0.00	0.61	0-4.04

ME, microscopic tumor extension; SD, Standard deviation; Other abbreviations are the same as **Table 1**.

### 3.2 Radio-pathological correlations

**Figure 2:**
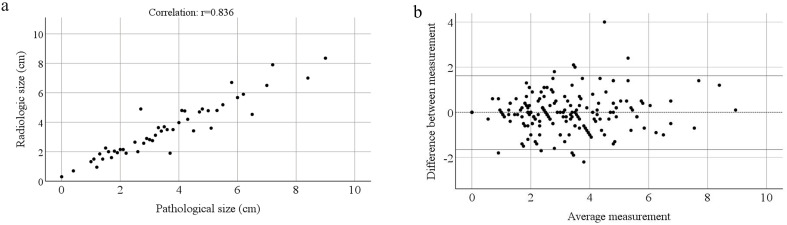
Consistency test of radiologic and pathological size of tumors. **(a)** Correlation between radiologic size and pathological size on all samples (without considering microscopic tumor extension). **(b)** Bland-Altmann plots of radiologic size and pathological size of tumors.

Comparative analysis of tumor size between radiography and pathology revealed a significant correlation (p<0.001) (**Figure 2A**) without considering the ME value. A Bland–Altman plot was constructed to evaluate the consistency between the radiological and pathological sizes of the tumors (**Figure 2B**).

### 3.3 Clinicopathological factors associated with ME

The specific results of the univariate and multivariate analyses are displayed in **Table 3**. The clinicopathologic factors correlated with ME distance involved pathologic type, tumor grade, and preoperative treatment method (all p < 0.05). In multivariate analysis, each of the above factors maintained significant independent correlation with ME distance, with a higher β-coefficient observed for the pathological type and preoperative treatment methods. Therefore, we determined the CTV margins based on the pathological type and preoperative treatment methods of NSCLC.

**Table 3:** Variables associated with microscopic tumor extension (ME) distance in univariate and multivariate linear regression model.

Variables	Univariate analysis			Multivariate analysis			
	B (95% CI)	β	P value	B (95% CI)	β	P value	VIF
Age (years)	-0.005 (-0.027 to 0.017)	-0.019	0.677				
Smoking							
Yes vs. No	-0.209 (-0.559 to 0.140)	-0.052	0.240				
Sex							
Male vs. Female	-0.009 (-0.404 to 0.385)	-0.002	0.962				
Pathologic types							
ADC vs. SCC	-0.969 (-1.302 to -0.636)	-0.247	<0.001	-0.805 (-1.142 to -0.469)	-0.205	<0.001	1.091
Tumor stage							
II vs. III	-0.517 (-0.861 to -0.173)	-0.131	0.003	-0.441 (-0.776 to -0.107)	-0.111	0.010	1.061
Treatment methods	-0.671 (-0.870 to -0.472)	-0.283	<0.001	-0.501 (-0.706 to -0.295)	-0.211	<0.001	1.120
DS vs. NAC	-0.464 (-0.880 to -0.048)	-0.109	0.029	-0.338 (-0.749 to 0.072)	-0.080	0.106	1.392
DS vs. NACI	-1.332 (-1.730 to -0.934)	-0.328	<0.001	-0.996 (-1.407 to -0.585)	-0.245	<0.001	1.524

VIF, variance inflation factor; Other abbreviations are the same as **Table 1**.

### 3.4 Direct surgery

**Figure 3:**
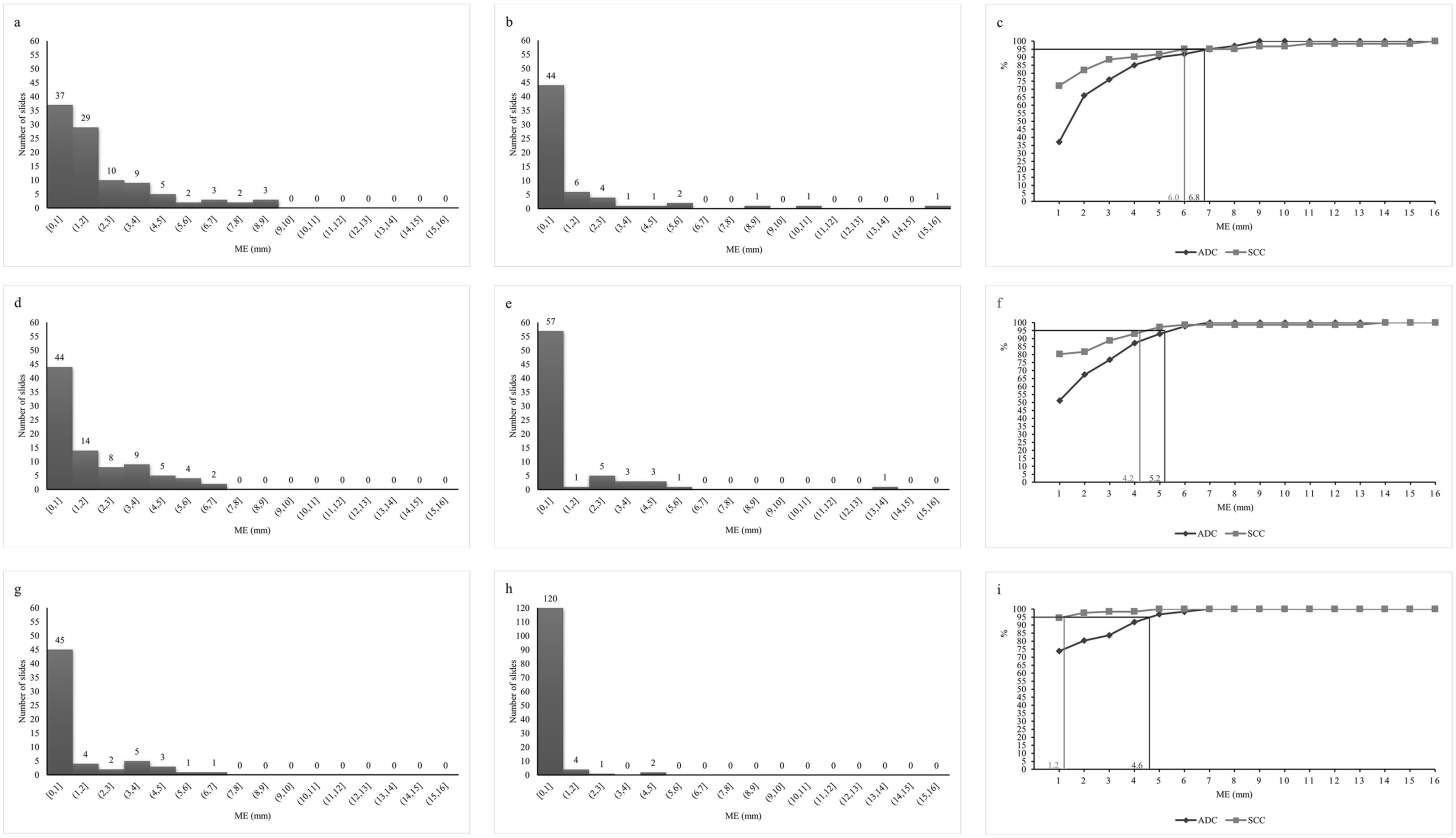
The distribution of microscopic tumor extension in each group. Microscopic tumor extension (ME) distribution of adenocarcinoma (ADC) **(a)** and squamous cell carcinomas (SCC) **(b)** in the direct surgery group. **(c)** Cumulative distribution of ME in ADC and SCC in the direct surgery group. ME distribution of ADC **(d)** and SCC **(e)** in the preoperative neoadjuvant chemotherapy group. **(f)** Cumulative distribution of ME in ADC and SCC in the preoperative neoadjuvant chemotherapy group. ME distribution of ADC **(g)** and SCC **(h)** in the preoperative neoadjuvant chemotherapy combined with immunotherapy group. **(i)** Cumulative distribution of ME in ADC and SCC in the preoperative neoadjuvant chemotherapy combined with immunotherapy group.

For direct surgery, we observed that the ME distance was significantly different between ADC and SCC (**Table 2**), with a mean of 1.98 mm (standard deviation [SD] 2.15; minimum [min] = 0; maximum [max] = 8.67) for ADC and 1.26 mm (SD 2.81; min=0; max=15.30) for SCC (p<0.001). We confirmed the differences between the two pathological types through analysis of the distribution of ME (**Supplementary Table S1**). The median ME distance for ADC and SCC were 1.38 mm and 0 mm, respectively. The ME frequency tables were classified in increments of 1 mm (**Figures 3A, B**); the ME cumulative frequency table is shown in **Figure 3C**. We concluded that for untreated patients with NSCLC to fully cover 95% of the ME, the GTV for ADC and SCC should be expanded by 6.80 mm and 6.00 mm to the CTV, respectively.

### 3.5 Preoperative neoadjuvant chemotherapy

For preoperative neoadjuvant chemotherapy, we observed that the ME distance was significantly different between ADC and SCC (**Table 2**), with a mean of 1.58 mm (SD 1.86; min=0; max=6.66) for ADC and 0.84 mm (SD 2.05; min=0; max=13.70) for SCC (p<0.001). We confirmed the differences between the two pathological types through analysis of the distribution of ME (**Supplementary Table S2**). The median ME distance for ADC and SCC were 0.79 mm and 0 mm, respectively. The ME frequency tables were classified in increments of 1 mm (**Figures 3D**, **3E**); the ME cumulative frequency table is shown in **Figure 3F**. We concluded that for patients with NSCLC who have received chemotherapy to fully cover 95% of the ME, the GTV for ADC and SCC should be expanded by 5.20 mm and 4.20 mm to the CTV, respectively.

### 3.6 Preoperative neoadjuvant chemotherapy combined with immunotherapy

For preoperative neoadjuvant chemotherapy combined with immunotherapy, we observed that the ME distance was significantly different between ADC and SCC (**Table 2**), with a mean of 0.87 mm (SD 1.64; min=0; max=6.33) for ADC and 0.14 mm (SD 0.61; min=0; max=4.04) for SCC (p<0.001). We confirmed the differences between the two pathological types through analysis of the distribution of ME (**Supplementary Table S3**). The median ME distance for both ADC and SCC was 0 mm. The ME frequency tables were classified in increments of 1 mm (**Figures 3G**, **H**); the ME cumulative frequency table is shown in **Figure 3I**. We concluded that for patients with NSCLC who have received chemotherapy combined with immunotherapy to fully cover 95% of the ME, the GTV for ADC and SCC should be expanded by 4.60 mm and 1.20 mm to the CTV, respectively.

## 4 Discussion

ME is considered as the “gold standard” in radiotherapy for accurately defining the CTV (17). Previous studies have quantitatively evaluated the subclinical invasion of primary lesions into surrounding tissues in untreated NSCLC to plan external beam radiotherapy (4, 18–20). However, to date, there is extremely little data on the ME of NSCLC after neoadjuvant therapy, which has resulted in a lack of unified and standardized guidelines to recommend the delineation of CTV for patients with NSLCL, who have received chemotherapy or chemotherapy combined with immunotherapy.

To the best of our knowledge, this is the first time in literature, that the macro pathology of NSCLC after chemotherapy and chemotherapy combined with immunotherapy was analyzed and ME distance beyond distinguishable GTV margins quantified. There is limited data available to directly determine whether CT tumor size fully represents gross pathological tumor size in NSCLC (18). To ensure the feasibility of our methodology, we first analyzed the relationship between tumor size measured using imaging and pathology. We confirmed that radiologic tumor size was closely related to macro pathological size without ME, similar to the conclusion of previous studies (4, 18, 19).

Several previous articles have published the ME values or the optimal CTV margins related to lung cancer radiotherapy. Giraud et al. (4) showed that to cover 95% of ME, the GTV of ADC and SCC required an outward expansion margin of 8 mm and 6 mm, respectively. Li et al. (20) determined that in order to ensure coverage of 95% of the ME when depicting the CTV, the required margins were 7 mm and 5 mm for ADC and SCC, respectively. ME measurements in these two studies were conducted using a light microscope. In our study, ME was accurately measured using a digital microscope, which may account for the discrepancy between the ME in the DS group and their research results. Schmitt et al. (18) after analyzing patients with NSCLC with localized stage T1N0 or T2aN0 who had undergone surgical treatment, concluded that to achieve 95% ME coverage, the required margins for SCC and ADC were 4.4 mm and 2.9 mm, respectively. Notably, in the above study, patients did not receive any other antitumor treatment before surgery; therefore, their ME range may not be suitable for inferring the CTV after receiving chemotherapy or chemotherapy combined with immunotherapy. Charlie et al. (19) included patients with NSCLC receiving NAC in their study and found that 95% of the ADC receiving and not receiving NAC had ME less than 5.3 mm (6.5 mm in the subgroup not receiving NAC), and 95% of the SCC receiving and not receiving NAC had ME less than 3.5 mm (3.7 mm in the subgroup not receiving NAC). Unfortunately, the number of patients receiving neoadjuvant chemotherapy included in their study was very small, only 28 cases, and there was no separate analysis of ME for this group of patients. Hu et al. (21) prospectively enrolled eight patients with small cell lung cancer (SCLC) and found that in patients who did or did not undergo NAC, a margin of 1.4 mm and 10.2 mm could cover 95% of ME, respectively. However, their research was restricted to limited-stage SCLC; thus, their conclusions were not applicable to NSCLC.

Regarding ME of tumors, the present study determined that the distribution of ME varied among different individuals with NSCLC, which correlated with the tumor pathological type, tumor stage, and preoperative treatment methods. It is noteworthy that the pathological type and preoperative treatment methods were the two major factors that significantly affected the ME distance. Hence, we defined the margin of CTV in NSCLC based on treatment methods and pathological types. To cover 95% of subclinical lesions, 6.80 mm and 6.00 mm CTV margins are recommended for direct surgical ADC and SCC, 5.20 mm and 4.20 mm CTV margins are recommended for ADC and SCC receiving chemotherapy before radiotherapy, and 4.60 mm and 1.20 mm CTV margins are recommended for ADC and SCC receiving chemotherapy combined with immunotherapy before radiotherapy.

Our research results indicate that there is a substantial difference in the microscopic infiltration range between ADC and SCC, with ADC demonstrating a larger microscopic infiltration range than that of SCC, consistent with the findings reported by Giraud et al. (4). Kara et al. (22) revealed that the infiltration of tumor cells in the central bronchus is more common in SCC than in ADC. However, the infiltration range of SCC is smaller than that of ADC, which may be correlated with the more invasive biological characteristics of ADC. Angela et al. (23) highlighted that spread through air spaces, mainly observed in lung ADC, has been identified as a novel mechanism of invasion, and which may be one of the reasons for the difference in ME between ADC and SCC. We also found that the mean tumor size of patients with SCC who did not undergo preoperative treatment was larger than that of patients with ADC using both imaging and pathology, while patients with SCC who underwent preoperative neoadjuvant treatment had a smaller average tumor size than that of patients with ADC; moreover, after neoadjuvant treatment, the PCR rate of SCC was higher than that of patients with ADC. SCC is more sensitive to current neoadjuvant therapy strategies than is ADC, and its potential benefits may be better (24, 25). Furthermore, this study demonstrated that tumor staging is related to ME size, which is consistent with the differences in ME research results of Schmitt et al. and Giraud et al. on NSCLC in stages I and I–IV, respectively (4, 18). This study also concluded that the ME size is related to the preoperative treatment received by the patients. The ME distance of the NAC group is smaller than that of the DS group, which may be due to the fact that chemotherapy drugs kill tumor cells by inhibiting key molecules and necessary cellular structures during cell replication (26). The ME distance of the NACI group is smaller than that of the NAC group. This is because programmed death receptor 1 (PD-1) and programmed death ligand 1 (PD-L1) inhibitors bind to PD-1 and PD-L1, respectively, preventing their interaction, restoring the recognition and killing effect of immune cells, avoiding immune escape of tumor cells, and thereby exerting anti-tumor effects (27). On the other hand, chemotherapy drugs can enhance anti-tumor immune responses through various mechanisms and to some extent inhibit the formation of immunosuppressive microenvironments (28–31). Previous studies have reported that NAC has better survival benefits than surgery alone (5–9), whereas subsequent studies have confirmed that NACI has better efficacy than NAC (10–12), which is consistent with the conclusions drawn from our study.

This study has some limitations. First, after the surgical specimen is removed from the human body, the tissue size may change, leading to inaccurate ME measurements. The surgical specimens were not fixed by the perfusion method because there is currently no experimental technique in literature that can make lung tissue specimens that leave the human body, completely consistent in morphology and structure similar to that of those inside the human body. Second, our analysis did not control for confounding factors which may exist, such as the types of drugs used for neoadjuvant therapy, dosage, treatment cycle, and scope of specimen collection; thus, further exploration is needed in the future.

## 5 Conclusions

This research aimed to personalize the delineation of the CTV in precise radiotherapy, thereby increasing the radiation dose to tumors while reducing the radiation damage to normal tissues, which is the primary goal of precision radiotherapy. We measured ME in NSCLC using three different treatment methods to define the CTV for radiotherapy. We observed that the mean ME of samples after chemotherapy or chemotherapy combined with immunotherapy (especially the latter) was much lower than the commonly adopted CTV range and differed considerably between the two histological types we studied. Therefore, the CTV currently used in clinical practice may increase therapeutic toxicity in patients. In our study, the mean ME values of ADC and SCC in the DS group were 1.98 mm and 1.26 mm, respectively. The mean ME values of ADC and SCC in the NAC group were 1.58 mm and 0.84 mm, respectively. The mean ME values of ADC and SCC in the NACI group were 0.87 mm and 0.14 mm, respectively. In the more practical context of radiotherapy, these findings can be represented in terms of the probability in which the ME corresponds to a given margin, hinging on the acceptable error risk. In the case of accepting a 5% error risk, for example, it would be essential to apply a margin of 6.80 mm and 6.00 mm to the ADC and SCC of DS, respectively; a margin of 5.20 mm and 4.20 mm to the ADC and SCC receiving chemotherapy, respectively; and a margin of 4.60 mm and 1.20 mm to the ADC and SCC receiving chemotherapy combined with immunotherapy, respectively.

## Data Availability

The raw data supporting the conclusions of this article will be made available by the authors, without undue reservation.
