# Supplementary material for: Determining optimal clinical target volume margins based on microscopic tumor extension in patients with non-small-cell lung cancer undergoing chemotherapy or chemotherapy combined with immunotherapy
Source: Front Oncol. 2026 Jan 12;15:1503615. doi: 10.3389/fonc.2025.1503615 (PMC12832255; doi:10.3389/fonc.2025.1503615)
Supplement: Supplementary file 1 [file Table1.docx]

**Supplementary Tables**

**Supplementary Table S1.** Microscopic tumor extension (ME) distribution for adenocarcinoma (A) and squamous cell carcinomas (B) in the direct surgery group.

| ME (mm) | Number | Cumulative | %Number | Cumulative% |
| --- | --- | --- | --- | --- |
| 0.20 | 24 | 24 | 24.00 | 24.00 |
| 0.40 | 6 | 30 | 6.00 | 30.00 |
| 0.60 | 2 | 32 | 2.00 | 32.00 |
| 0.80 | 3 | 35 | 3.00 | 35.00 |
| 1.00 | 2 | 37 | 2.00 | 37.00 |
| 1.20 | 7 | 44 | 7.00 | 44.00 |
| 1.40 | 7 | 51 | 7.00 | 51.00 |
| 1.60 | 7 | 58 | 7.00 | 58.00 |
| 1.80 | 3 | 61 | 3.00 | 61.00 |
| 2.00 | 5 | 66 | 5.00 | 66.00 |
| 2.20 | 1 | 67 | 1.00 | 67.00 |
| 2.40 | 3 | 70 | 3.00 | 70.00 |
| 2.60 | 5 | 75 | 5.00 | 75.00 |
| 2.80 | 1 | 76 | 1.00 | 76.00 |
| 3.20 | 2 | 78 | 2.00 | 78.00 |
| 3.40 | 1 | 79 | 1.00 | 79.00 |
| 3.80 | 4 | 83 | 4.00 | 83.00 |
| 4.00 | 2 | 85 | 2.00 | 85.00 |
| 4.20 | 1 | 86 | 1.00 | 86.00 |
| 4.40 | 1 | 87 | 1.00 | 87.00 |
| 4.60 | 2 | 89 | 2.00 | 89.00 |
| 4.80 | 1 | 90 | 1.00 | 90.00 |
| 5.20 | 1 | 91 | 1.00 | 91.00 |
| 5.40 | 1 | 92 | 1.00 | 92.00 |
| 6.60 | 1 | 93 | 1.00 | 93.00 |
| 6.80 | 2 | 95 | 2.00 | 95.00 |
| 7.20 | 1 | 96 | 1.00 | 96.00 |
| 7.80 | 1 | 97 | 1.00 | 97.00 |
| 8.40 | 2 | 99 | 2.00 | 99.00 |
| 8.80 | 1 | 100 | 1.00 | 100.00 |

A

| ME (mm) | Number | Cumulative | %Number | Cumulative% |
| --- | --- | --- | --- | --- |
| 0.20 | 39 | 39 | 63.93 | 63.93 |
| 0.40 | 1 | 40 | 1.64 | 65.57 |
| 0.60 | 1 | 41 | 1.64 | 67.21 |
| 1.00 | 3 | 44 | 4.92 | 72.13 |
| 1.20 | 1 | 45 | 1.64 | 73.77 |
| 1.40 | 2 | 47 | 3.28 | 77.05 |
| 1.80 | 2 | 49 | 3.28 | 80.33 |
| 2.00 | 1 | 50 | 1.64 | 81.97 |
| 2.20 | 1 | 51 | 1.64 | 83.61 |
| 2.60 | 2 | 53 | 3.28 | 86.89 |
| 3.00 | 1 | 54 | 1.64 | 88.52 |
| 3.80 | 1 | 55 | 1.64 | 90.16 |
| 4.40 | 1 | 56 | 1.64 | 91.80 |
| 5.80 | 1 | 57 | 1.64 | 93.44 |
| 6.00 | 1 | 58 | 1.64 | 95.08 |
| 8.80 | 1 | 59 | 1.64 | 96.72 |
| 11.00 | 1 | 60 | 1.64 | 98.36 |
| 15.40 | 1 | 61 | 1.64 | 100.00 |

B

**Supplementary Table S2.** Microscopic tumor extension (ME) distribution for adenocarcinoma (A) and squamous cell carcinomas (B) in the preoperative neoadjuvant chemotherapy group.

| ME (mm) | Number | Cumulative | %Number | Cumulative% |
| --- | --- | --- | --- | --- |
| 0.20 | 37 | 37 | 43.02 | 43.02 |
| 0.60 | 3 | 40 | 3.49 | 46.51 |
| 0.80 | 3 | 43 | 3.49 | 50.00 |
| 1.00 | 1 | 44 | 1.16 | 51.16 |
| 1.20 | 2 | 46 | 2.33 | 53.49 |
| 1.40 | 3 | 49 | 3.49 | 56.98 |
| 1.60 | 2 | 51 | 2.33 | 59.30 |
| 1.80 | 4 | 55 | 4.65 | 63.95 |
| 2.00 | 3 | 58 | 3.49 | 67.44 |
| 2.40 | 3 | 61 | 3.49 | 70.93 |
| 2.60 | 3 | 64 | 3.49 | 74.42 |
| 2.80 | 1 | 65 | 1.16 | 75.58 |
| 3.00 | 1 | 66 | 1.16 | 76.74 |
| 3.20 | 1 | 67 | 1.16 | 77.91 |
| 3.40 | 1 | 68 | 1.16 | 79.07 |
| 3.60 | 2 | 70 | 2.33 | 81.40 |
| 3.80 | 1 | 71 | 1.16 | 82.56 |
| 4.00 | 4 | 75 | 4.65 | 87.21 |
| 4.20 | 2 | 77 | 2.33 | 89.53 |
| 4.40 | 2 | 79 | 2.33 | 91.86 |
| 4.80 | 1 | 80 | 1.16 | 93.02 |
| 5.20 | 2 | 82 | 2.33 | 95.35 |
| 5.80 | 1 | 83 | 1.16 | 96.51 |
| 6.00 | 1 | 84 | 1.16 | 97.67 |
| 6.60 | 1 | 85 | 1.16 | 98.84 |
| 6.80 | 1 | 86 | 1.16 | 100.00 |

A

| ME (mm) | Number | Cumulative | %Number | Cumulative% |
| --- | --- | --- | --- | --- |
| 0.20 | 53 | 53 | 74.65 | 74.65 |
| 0.60 | 2 | 55 | 2.82 | 77.46 |
| 0.80 | 1 | 56 | 1.41 | 78.87 |
| 1.00 | 1 | 57 | 1.41 | 80.28 |
| 1.40 | 1 | 58 | 1.41 | 81.69 |
| 2.40 | 2 | 60 | 2.82 | 84.51 |
| 2.80 | 1 | 61 | 1.41 | 85.92 |
| 3.00 | 2 | 63 | 2.82 | 88.73 |
| 3.60 | 1 | 64 | 1.41 | 90.14 |
| 3.80 | 1 | 65 | 1.41 | 91.55 |
| 4.00 | 1 | 66 | 1.41 | 92.96 |
| 4.20 | 3 | 69 | 4.23 | 97.18 |
| 5.20 | 1 | 70 | 1.41 | 98.59 |
| 13.80 | 1 | 71 | 1.41 | 100.00 |

B

**Supplementary Table S3.** Microscopic tumor extension (ME) distribution for adenocarcinoma (A) and squamous cell carcinomas (B) in the preoperative neoadjuvant chemotherapy combined with immunotherapy group.

| ME (mm) | Number | Cumulative | %Number | Cumulative% |
| --- | --- | --- | --- | --- |
| 0.20 | 44 | 44 | 72.13 | 72.13 |
| 0.40 | 1 | 45 | 1.64 | 73.77 |
| 1.20 | 1 | 46 | 1.64 | 75.41 |
| 1.40 | 1 | 47 | 1.64 | 77.05 |
| 1.60 | 1 | 48 | 1.64 | 78.69 |
| 2.00 | 1 | 49 | 1.64 | 80.33 |
| 2.20 | 1 | 50 | 1.64 | 81.97 |
| 2.40 | 1 | 51 | 1.64 | 83.61 |
| 3.40 | 2 | 53 | 3.28 | 86.89 |
| 3.60 | 3 | 56 | 4.92 | 91.80 |
| 4.40 | 1 | 57 | 1.64 | 93.44 |
| 4.60 | 1 | 58 | 1.64 | 95.08 |
| 4.80 | 1 | 59 | 1.64 | 96.72 |
| 5.40 | 1 | 60 | 1.64 | 98.36 |
| 6.40 | 1 | 61 | 1.64 | 100.00 |

A

| ME (mm) | Number | Cumulative | %Number | Cumulative% |
| --- | --- | --- | --- | --- |
| 0.20 | 118 | 118 | 92.91 | 92.91 |
| 0.40 | 1 | 119 | 0.79 | 93.70 |
| 1.00 | 1 | 120 | 0.79 | 94.49 |
| 1.20 | 1 | 121 | 0.79 | 95.28 |
| 1.60 | 1 | 122 | 0.79 | 96.06 |
| 2.00 | 2 | 124 | 1.57 | 97.64 |
| 2.40 | 1 | 125 | 0.79 | 98.43 |
| 4.20 | 2 | 127 | 1.57 | 100.00 |

B
